# Recent Development in ITO-free Flexible Polymer Solar Cells

**DOI:** 10.3390/polym10010005

**Published:** 2017-12-22

**Authors:** Shudi Lu, Yang Sun, Kuankuan Ren, Kong Liu, Zhijie Wang, Shengchun Qu

**Affiliations:** 1 Department of Physics, Hebei Normal University of Science & Technology, Qinhuangdao 066004, China; 2 Key Laboratory of Semiconductor Materials Science, Beijing Key Laboratory of Low Dimensional Semiconductor Materials and Devices, Institute of Semiconductors, Chinese Academy of Sciences, Beijing 100083, China; syang@semi.ac.cn (Y.S.); renkk1991@semi.ac.cn (K.R.); liukong@semi.ac.cn (K.L.)

**Keywords:** polymer solar cells, transparent electrodes, performance, flexibility, stability

## Abstract

Polymer solar cells have shown good prospect for development due to their advantages of low-cost, light-weight, solution processable fabrication, and mechanical flexibility. Their compatibility with the industrial roll-to-roll manufacturing process makes it superior to other kind of solar cells. Normally, indium tin oxide (ITO) is adopted as the transparent electrode in polymer solar cells, which combines good conductivity and transparency. However, some intrinsic weaknesses of ITO restrict its large scale applications in the future, including a high fabrication price using high temperature vacuum deposition method, scarcity of indium, brittleness and scaling up of resistance with the increase of area. Some substitutes to ITO have emerged in recent years, which can be used in flexible polymer solar cells. This article provides the review on recent progress using other transparent electrodes, including carbon nanotubes, graphene, metal nanowires and nanogrids, conductive polymer, and some other electrodes. Device stability is also discussed briefly.

## 1. Introduction

With the growing energy demand worldwide, utilization of clean and renewable energy sources, such as solar energy, has attracted extensive attention in the past decades. Solar cells, which can convert solar energy to electricity, have realized industry fabrication and large scale application, especially for the silicon based solar cells [1,2]. At the same time, other types of solar cells have also emerged, with thin film photovoltaic devices being a hotspot in the recent years. Among them, polymer solar cells have been studied, broadly owing to their advantages of light-weight, low-cost, ease of fabrication, and mechanical flexibility [3,4,5,6,7,8,9,10,11,12,13]. The power conversion efficiency (PCE) of polymer solar cells has exceeded 10% for single-junction solar cells [14,15,16], and the result is even higher for tandem and multi-junction devices [17,18]. Normally, highly efficient polymer solar cells are fabricated on rigid indium tin oxide (ITO) glass substrates in laboratory. While considering the large scale manufacturing process in industry, where a roll-to-roll method is preferred, research about flexible polymer solar cells is of great importance, and tremendous efforts have been devoted into this research area. ITO on flexible substrates like polyethyleneterphthalate (PET) and polyimide (PI) possesses good optical and electrical properties, and the corresponding devices are capable of getting comparable efficiency to that of the ITO glass counterparts [19,20,21]. However, some intrinsic drawbacks of ITO make it not the ideal choice for flexible transparent electrode in polymer solar cells. As it is commonly known, indium is rare metal with limited production per year, while the large demand from display industry for usage as transparent electrodes caused the rise of indium price [22]. Moreover, ITO is typically fabricated through expensive high-temperature–vacuum-deposition method. It is revealed from the life cycle analyses that ITO accounts for fifty percent of the total cost of organic photovoltaic devices, which is an obstacle for low-cost fabrication [23]. Besides, there is a degradation problem for ITO when in contact with the commonly used acidic PEDOT:PSS buffer layer [24,25]. There is another concern that ITO is brittle that cannot stand mechanical deformation. Therefore, finding substitutes to ITO is of great significance. In the recent years, several kinds of alternative transparent electrodes have been adopted in polymer solar cells, including carbon nanotubes, graphene, metal nanowires and nanogrids, conductive polymers, and so on. These electrodes can be fabricated either on rigid or flexible substrates [22,26,27,28].

In this review, we focus on flexible polymer solar cells using different transparent conductive electrodes other than ITO. The major research progress and recent research results are summarized mainly from two aspects. In one aspect, the properties of the flexible conductive electrode are given, including the sheet resistance (*R*s), optical transmittance, as well as the mechanical flexibility of the electrode films. In another aspect, the performance of the resulting flexible polymer solar cells that are based on these electrodes is listed, focusing on the PCEs and flexibility. Besides, the stability of the devices, which is critical for practical applications, is also discussed.

## 2. Alternative Transparent Electrodes to ITO

For the development of ITO-free polymer solar cells, we will concentrate our attention on the alternative conductive electrodes. The following discussion is conducted according to different electrode materials, which are carbon nanotubes, graphene, metal nanowires and nanogrids, conductive polymer, and some other transparent electrodes. Performance of the flexible polymer solar cells is summarized accordingly.

### 2.1. Carbon Nanotubes

In 1991, Sumio Iijima observed a new type of carbon structure with needle-like tubes, which was named carbon nanotube [29]. Widespread research has been conducted since then, and this material also gains broad commercial interest with over several thousand tons production per year, carbon nanotubes based products include lithium ion batteries, water filters, and so on [30].

The usage of carbon nanotubes as transparent conductive film started from 2004, and they have shown promising application trend in optoelectronic devices, including solar cells, organic light-emitting diodes (OLEDs), and touch panels [31]. Carbon nanotubes have been adopted in several kinds of solar cells, including polymer solar cells [32], dye-sensitized solar cells [33,34], and perovskite solar cells [35,36], either as transparent electrodes or as hole-transport layers. They have also been added to the active layers of polymer solar cells to enhance device efficiency [37,38,39].

Both single-walled carbon nanotubes (SWCNTs) and multi-walled carbon nanotubes (MWCNTs) have been applied. The structure of SWCNTs can be described as the rolling of a planar graphene sheet. When multi-layers formed during the fabrication process, they are called MWCNTs.

The wide spread research of carbon nanotubes is based on their good properties, such as good chemical stability, mechanical flexibility, relatively low sheet resistance, and high visible light transmittance. Several wet or dry fabrication methods of carbon nanotubes have been developed, and continuous preparation of carbon nanotubes (CNT) thin films has also been realized.

**Figure 1:**
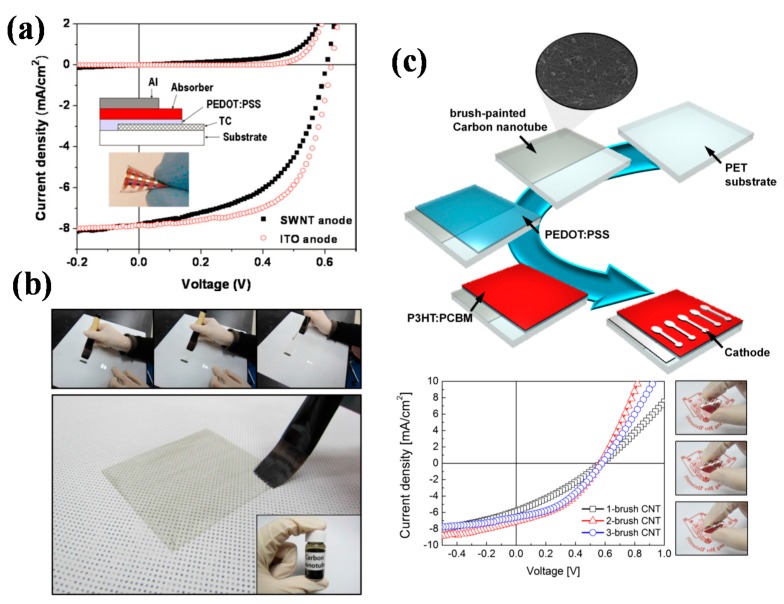
(**a**) Current density–voltage (J–V) characteristics of P3HT:PCBM devices under AM1.5G conditions using indium tin oxide (ITO) on glass and flexible SWNTs on PET as the anodes, respectively. Reprinted from [40], with the permission of AIP Publishing; (**b**) Picture of brush printing process (upper panels) to fabricate transparent carbon nanotubes (CNT) network electrodes and transparent 3-brush CNT electrode; (**c**) Schematic of the fabrication process for bulk-heterojunction OSCs on a CNT network electrode and J–V curves of FOSCs (flexible organic solar cells) fabricated on the CNT electrodes where the right panel shows the flexibility of the FOSCs with a brush-painted CNT network electrode. Reprinted from [41], Copyright 2014, with permission from Elsevier.

Michael et al. adopted a transfer-printing method to produce SWNT (single-walled carbon nanotube) films on flexible PET substrates as transparent electrodes for polymer solar cells with structure of PET/SWNTs/PEDOT:PSS/P3HT:PCBM/Al [40]. SWNTs that were used here were produced by arc discharge, and are commercially available. The SWNT electrodes were relatively homogeneous and had a low roughness, with sheet resistance (*R*s) of 200 Ω/sq and transmittance of 85% at 550 nm. The corresponding solar cells exhibited a PCE of 2.5%, close to the glass/ITO counterparts (3%) (Figure 1a). Bending tests showed no efficiency degradation when the devices were folded to radii of curvature of 5 mm, indicating an excellent flexibility of the SWNT electrodes [40].

The fabrication method of carbon nanotubes electrode is a key factor influencing the large-scale application of carbon nanotubes based solar cells, low-cost and simple process is essential. Da-Young et al. prepared carbon nanotube electrode through a simple brush-painting method (Figure 1b) [41]. The electrode could be directly painted on flexible PET substrate from carbon nanotube inks, which were purchased from Top Nanosis, resulting in good optical, electrical, and mechanical performance, with *R*s of 286 Ω/sq and transmittance of 78.45%. The corresponding flexible polymer solar cells achieved a PCE of 1.632% (Figure 1c). Notably, the devices showed excellent stability against various bending modes. The brush-painting method provided a convenient and cost-effective fabrication process for carbon nanotube based solar cells, which is also applicable to industry production [41].

Pristine carbon nanotubes cannot always meet the requirements of various devices, to improve their properties, modification with dopants or hybridization with other materials has been proved to be an effective way [31].

**Figure 2:**
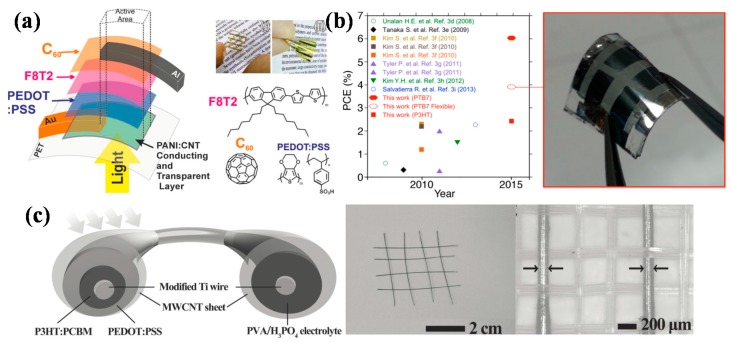
(**a**) The configuration of the solar cell built over a PET/PANI:CNT5 film, and the corresponding chemical structures of the organic layers. The photographs show the PET/PANI:CNT5 film (I) and the flexible solar cell (II). Reproduced with permission from [43]; published by John Wiley and Sons, 2013. (**b**) Reported PCEs of CNT organic solar cells (OSCs) on glass (closed symbols) and on flexible substrate (open symbols) (**left**) and a picture of the present PET-based flexible SWCNT OSC (**right**). Reprinted with permission from [44]. Copyright 2015 American Chemical Society. (**c**) Schematic illustration to the structure of all-solid-state, coaxial and integrated fiber device, corresponding to the PC and ES parts (**left**). “Energy fibers” being woven into a textile structure with each other and photograph of two “energy fibers” being woven into a flexible aramid fibers textile (**right**). Reproduced with permission from [46]; published by John Wiley and Sons, 2014.

Rodrigo et al. reported the synthesis and application as transparent anode of polyaniline/carbon nanotubes composites thin films (PANI:CNT). HiPco (high-pressure carbon monoxide) purified SWNTs were firstly modified by DNA and forming the composite electrode through an in situ polymerization process. Combination of polyaniline and carbon nanotubes showed superior property when compared with pure polyaniline or carbon nanotubes. Incorporation of carbon nanotubes improved the conductivity and stability of polyaniline film. In addition, polyaniline acted as conductive glue between carbon nanotubes, solving the problem of high resistance between tubes and resulting in improved conductivity [42]. The composites films exhibited a relatively high transmittance of 89% at 550 nm with *R*s of 295 Ω/sq. Polymer solar cell with structure of PET/PANI:CNT/PEDOT:PSS/F8T2/C60/Al (Figure 2a) achieved a PCE of 2.27%, surpassing the commercial Glass/ITO and Glass/FTO based devices [43]. By using MoO*_x_* doped SWCNT films as transparent electrode, Jeon et al. demonstrated a flexible polymer solar cell with PCE of 3.91% (Figure 2b). Doping can lower the resistivity and increase the transmittance of the SWCNT films, which is essential for solar cell efficiency improvement [44]. These results indicate that the modification or doping of carbon nanotube films can be an effective way to improve the performance. Carbon nanotubes can also be combined with other conductive materials, like metal nanowires or conducting polymers, to get better performance. It is reported that carbon nanotube/silver nanowire hybrid electrode on PET substrate obtained *R*s as low as 20.9–53.9 Ω/sq, with high transmittance of 84–91%. The performance was comparable to the commonly used ITO electrode [45].

Despite the normal planar thin film polymer solar cells, other architectures, like fiber or wire-shaped devices using carbon nanotubes as electrode, have also appeared. Zhang et al. designed a flexible “energy fiber” which was fiber-shaped device integrating photovoltaic conversion and energy storage (Figure 2c). The photovoltaic part was similar to the normal polymer solar cells, except in a wire format. The structure was designed as Ti wire/TiO_2_ nanotubes/P3HT:PCBM/PEDOT:PSS/MWCNT, in which Ti wire and MWCNT sheet served as the two electrodes. The MWCNT sheet electrode was produced from MWCNT arrays, which were fabricated through chemical vapor deposition method. The photovoltaic conversion part got a PCE of 1.01%, though it was relatively low compared with normal polymer solar cells, this “energy fiber” could combine the function of electricity generation and storage, and it showed excellent flexibility. The “energy fiber” could be bent into various shapes or even woven with each other, and it could maintain over 90% of the photoelectric conversion and storage efficiency after bending for 1000 cycles. The good conductivity and flexibility of MWCNT is a guarantee for the device performance. The results provide a new direction for the design of flexible solar cells and other photoelectric devices [46].

Carbon nanotube is a promising candidate for transparent electrode in polymer solar cells, but its overall properties still lag behind the commercial ITO. There are several aspects that need to be concerned. For example, the conductivity and transparency need to be further improved, and surface roughness is still a hinder to good performance. More studies in the future will help to pave the way towards its large-scale application in multiple devices.

### 2.2. Graphene

Since K. S. Novoselov et al. reported the fabrication of graphene using simple mechanical exfoliation method [47], research on graphene has been a hotspot, and the corresponding material and device fabrication have attracted extensive interests of researchers. It has been applied in various flexible devices, including solar cells, fuel cells, supercapacitors, organic light emitting diodes, etc. [48,49]. Graphene is a zero bandgap semimetal with the valence band overlapping a little with the conduction band [47]. Its superior properties, including high carrier mobility (2 × 10^5^ cm^2^/Vs), high Young’s modulus (1 × 10^3^ GPa), large specific surface area (2.6 × 10^3^ m^2^/g), together with good transparency, have made it a good candidate for flexible transparent electrode [50].

Graphene thin films can be fabricated via several methods, including mechanical exfoliation, reduction of graphene oxide (rGO), chemical vapor deposition (CVD), and so on [51,52]. The mechanical exfoliation method can obtain graphene films with high quality, but it is normally used for lab study, not being suitable for large-scale production. CVD, rGO, and some other solution based methods are mostly studied in recent years, below we will list some representative results of the fabrication of graphene thin films and their application in polymer solar cells.

#### 2.2.1. rGO Method

**Figure 3:**
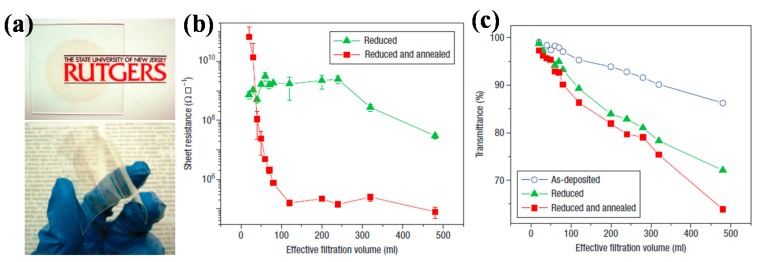
(**a**) Photographs of graphene oxide (GO) thin films on glass and plastic substrates; (**b**) Sheet resistance and (**c**) transmittance at 550 nm as a function of filtration volume for reduced GO thin films. Reproduced with permission from [53]; published by Nature Publishing Group, 2008.

The rGO method is relatively low-cost and can be applied to large-area fabrication. Goki Eda et al. reported the deposition of large-area uniform graphene films on glass and plastic substrates with thickness of 1–5 nm by reduction of graphene oxide (GO) (Figure 3a). A vacuum filtration method was used to fabricate GO films with controllable layers, by adjusting the GO suspension concentration and filtration volume, the desired film thickness could be obtained, with the thinnest film as low as 1–2 nm. Efficient reduction of GO films under a combination of hydrazine vapor exposure and low temperature annealing resulted in thin films with tunable sheet resistance over six orders of magnitude and transparency in the range of 60–95% (Figure 3b). This method is solution-based, and the resulting reduced GO films are uniform, making it promising for device application [53].

**Figure 4:**
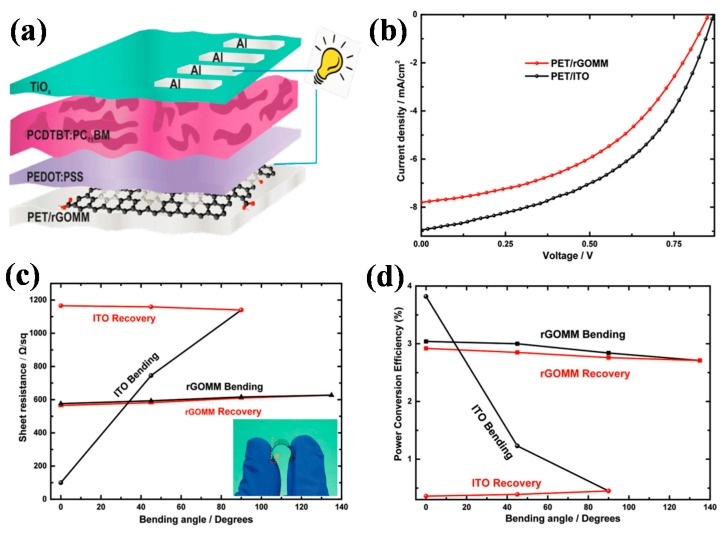
(**a**) Schematic illustration of BHJ OPV device with the laser-induced rGOMM as TCE; (**b**) The illuminated J–V curves of the solar cells with rGO micromesh (rGOMM) (red) and ITO (black) as TCE; (**c**) Sheet resistance versus device bending angle of rGOMM- and ITO-based OPV cells. The inset photo represents rGOMM-based device subjected to bending; (**d**) Power conversion efficiency of rGOMM- and ITO-based flexible devices under and after bending at certain angles. Reproduced with permission from [55]; published by John Wiley and Sons, 2015.

The reduction of GO films can be carried out through different processes. Kymakis et al. reported a reduction of GO thin films by using pulsed femtosecond laser beam. This nonthermal method reduced the GO films in situ on the flexible PET substrates, without the need for film transfer. Through tuning the pulse laser, parameters such as layer output power and laser numbers, the reduction rate as well as the sheet resistance of GO films could be well controlled. The whole process did not cause any damage to the flexible substrates. The resulting flexible GO films exhibited excellent flexibility, the sheet resistance change was smaller than 13% even when the bending angle was enlarged to 135°, making it a good choice for flexible transparent electrodes. Polymer solar cells based on this laser induced GO electrode with structure of PET/rGO/PEDOT:PSS/P3HT:PCBM/Al obtained an efficiency of 1.1%, which was claimed to be the highest result using rGO electrode at that time [54]. Two years later, the same group improved the efficiency of flexible polymer solar cells based on rGO micromesh (rGOMM) electrodes to 3.05%, with device structure of PET/rGOMM/PEDOT:PSS/PCDTBT:PC_71_BM/TiO_x_/Al (Figure 4a) [55]. The rGO micromesh was obtained by performing a laser pattering technique on rGO films. The periodicity and neck width of the meshes can be controlled automatically according to preprogrammed patterns. Sheet resistance and transparency of rGO layers were affected by the pit depth and there was a tradeoff between the two elements which can be optimized by tuning fabrication parameters, like laser pulses number. The rGOMM film showed superior transparency and conductivity as compared with the pristine rGO films. To be specific, rGO films with transparency of 20% and sheet resistance of 780 Ω/sq could result in rGOMM films with transparency of 59% and sheet resistance of 565 Ω/sq. Thus, the rGO based polymer solar cells also showed much lower efficiency than the rGOMM based devices. To be noteworthy, the rGOMM devices exhibited a comparable PCE with ITO based devices (3.82%) (Figure 4b) and good bending stability (Figure 4c,d). This result indicated that the rGOMM films were excellent candidates for flexible transparent electrodes that could be applied to various electronic devices.

The rGO method is a relatively low-cost process and has been broadly used for the fabrication of graphene films, whereas high density of defects and high sheet resistance of the films limited the device efficiency. Efforts have been paid to improve the rGO film performance, such as doping with carbon nanotubes, polymers, or inorganic nanoparticles to form composites, etc.

#### 2.2.2. CVD Method

CVD is another widely adopted method to fabricate graphene electrode, it is regarded as one of the most promising method to obtain high quality graphene films. The according flexible photovoltaic devices based on CVD-grown graphene electrode were able to obtain a high PCE of above 7%, showing the obvious advantages over other methods [56]. While the CVD method also has some disadvantages, like high-cost and the need for film transfer, which limits its application to some extent. The following are some recent developments regarding CVD-grown graphene films and their application in flexible polymer solar cells.

**Figure 5:**
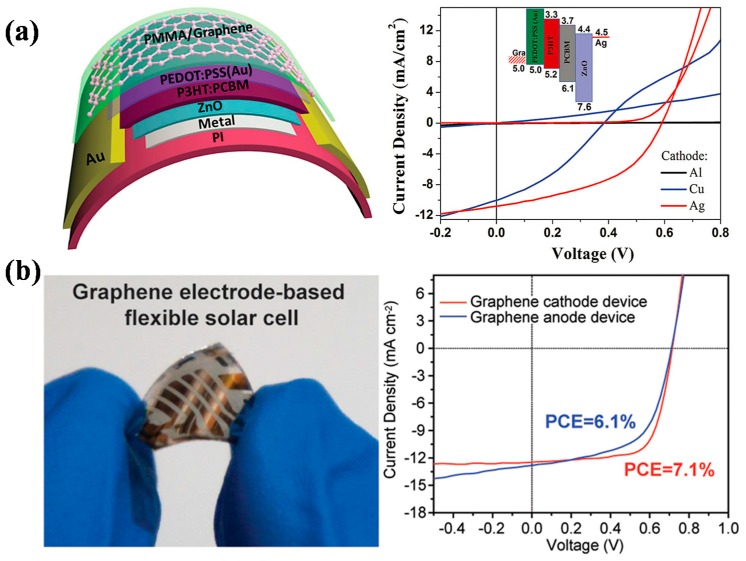
(**a**) Schematic diagram of an OPV with the inverted structure: PI/Metal/ZnO/P3HT:PCBM/PEDOT:PSS(Au)/Graphene/PMMA and J–V curves of the devices. Reproduced with permission from [57]; published by John Wiley and Sons, 2013. (**b**) Photograph of a graphene electrode-based flexible solar cell and the corresponding J–V characteristics of graphene cathode and anode based devices. Reprinted with permission from [56]. Copyright 2014 American Chemical Society.

Liu et al. fabricated flexible polymer solar cells using multilayer CVD graphene as top electrode (anode), the device exhibited a PCE of 3.2%, with structure of PI/Ag/ZnO/P3HT:PCBM/PEDOT:PSS(Au)/Graphene/PMMA. Au nanoparticles were doped into PEDOT:PSS solution by adding HAuCl_4_, forming PEDOT:PSS (Au) composite. This composite was applied to dope the bottom surface of graphene, and doping could obviously reduce the sheet resistance of graphene film, getting a low *R*s of 158 ± 30 Ω/sq for single layer graphene, and an even lower result of 68 ± 10 Ω/sq for four-layer graphene. Three metals (Al, Cu, Ag) were used as the bottom electrode, while Ag outperformed others, obtaining the highest PCE of 3.17% with double-layer graphene as top electrode (Figure 5a). The number of graphene layers also had an important effect on the performance of solar cells. Bending test showed that the device could maintain over 90% of PCE after bending for 1000 cycles, indicating the excellent flexibility of the devices. What is more, the multilayer graphene top electrode was able to protect the devices from air, eliminating the need for device packaging [57].

Graphene can be used as both anode and cathode in organic photovoltaics. Park et al. fabricated graphene films via low pressure CVD (LPCVD) method, and applied them as anode in conventional structured bi-layer small molecule solar cells and as cathode in inverted devices. By modifying graphene surface using double layer hole injection layers, PCE close to that of ITO based device was obtained [58]. Later, Park et al. demonstrated highly efficient flexible polymer solar cells by adopting graphene as anode or cathode, reaching record-high PCEs of 6.1% for graphene anode device and 7.1% for graphene cathode device (Figure 5b). The graphene electrode was synthesized via LPCVD method, and consisted of three monolayers. The resulting film had good conductivity and transparency, with *R*s of 300 Ω/sq and transmittance of 92%. Critical aspects contributing to the high device efficiency were demonstrated to be the thermal annealing of MoO_3_ layer and the direct deposition of ZnO layer on graphene by spin-coating. Bending results showed that the graphene based devices were robust under mechanical deformation, without any significant performance change after bending for 100 cycles at 5 mm radius [56].

**Figure 6:**
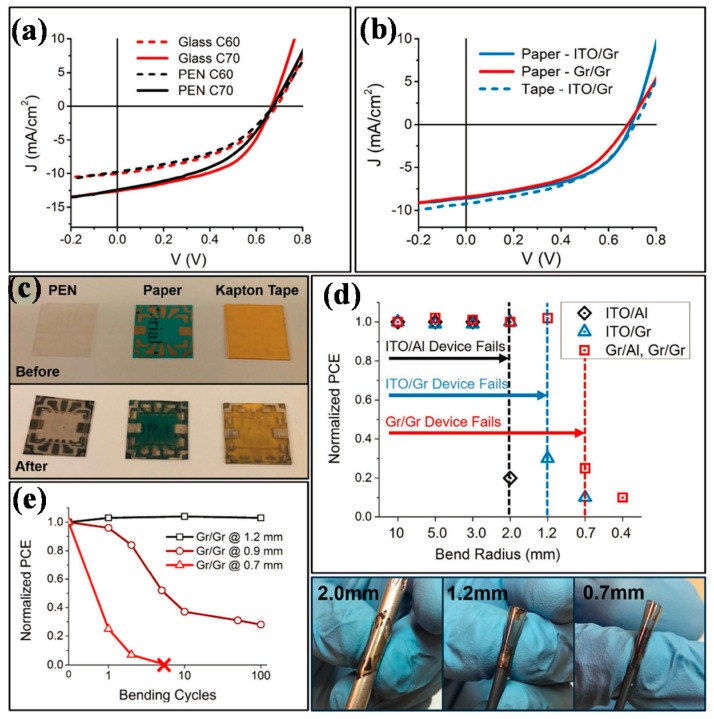
(**a**) Comparison of PC_60_BM and PC_70_BM Gr/Gr devices on rigid glass substrates and on flexible PEN substrates; (**b**) J–V curves of devices on paper and Kapton tape and (**c**) photographs of substrates (PEN, paper, Kapton tape) before and after device fabrication; (**d**) Device performances when bent to different radii of curvature and photographs of a device being bent; (**e**) Normalized PCE of Gr/Gr devices versus number of bending cycles. Reproduced with permission from [59]; published by John Wiley and Sons, 2016.

Polymer solar cells based on all graphene electrodes have also been reported. Song et al. fabricated flexible transparent polymer solar cells using LPCVD grown graphene as both anode and cathode, with device structure of Graphene/PEDOT:PSS/ZnO/PDTP-DFBT:PCBM/MoO_3_/Graphene. The bottom graphene film was transferred via commonly used PMMA method, while for the top graphene film, a room temperature dry transfer process was developed, which could be applied to flexible substrates. The devices had an optical transmittance of 61% in the visible light range. Solar cells based on all graphene electrodes reached an efficiency of 3.7% on flexible PEN substrates. Devices were also fabricated on paper and Kapton tape, indicating the feasibility of graphene based devices on different substrates, photographs of devices are shown in Figure 6. The devices could work under bending conditions as long as the radius of curvature was below a critical value [59].

Graphene can be combined with other materials to get better performance. An et al. adopted a combined film of graphene and one-dimensional Au nanograte (Gr/Au-NG) as transparent conducting electrode in flexible polymer solar cells, obtaining an efficiency of 6.38%, which is comparable to that of ITO based devices. Graphene was synthesized via CVD method, and transferred onto the flexible substrate embedded with Au-NG. The Gr/Au-NG hybrid films got a low sheet resistance of 8 and 19 Ω/sq, with Au-NG spacing of 500 and 1000 nm, respectively. The according transmittance was 78.3% and 81.5% at wavelength of 550 nm. The hybrid films also showed good stability under mechanical bending. The flexible devices using the Gr/Au-NG hybrid electrode had comparable PCE to that of ITO devices, while showing enhanced flexibility [60].

Besides rGO and CVD methods, there are also other methods being adopted to fabricate graphene films, which are applicable as transparent electrode in polymer solar cells. Lima et al. used a blend of GO and PEDOT:PSS as transparent electrode in a bilayer polymer solar cells with structure of PET/GO:PEDOT/F8T2/C60/Al device, and a PCE of 1.10% was achieved. The usage of pure GO instead of rGO simplified the fabrication procedure, demanding for fewer steps. The GO:PEDOT composite did not need any surfactants or dopants, and they also played the role as hole transport materials in the device. By tuning the ratio of GO and PEDOT:PSS, optical transmittance and sheet resistance of the films could be controlled. The films also showed good flexibility, without any decrease in conductivity after bending for 1000 cycles. This new approach was compatible with large scale fabrication process, providing a new approach for graphene based organic photovoltaics [61].

**Figure 7:**
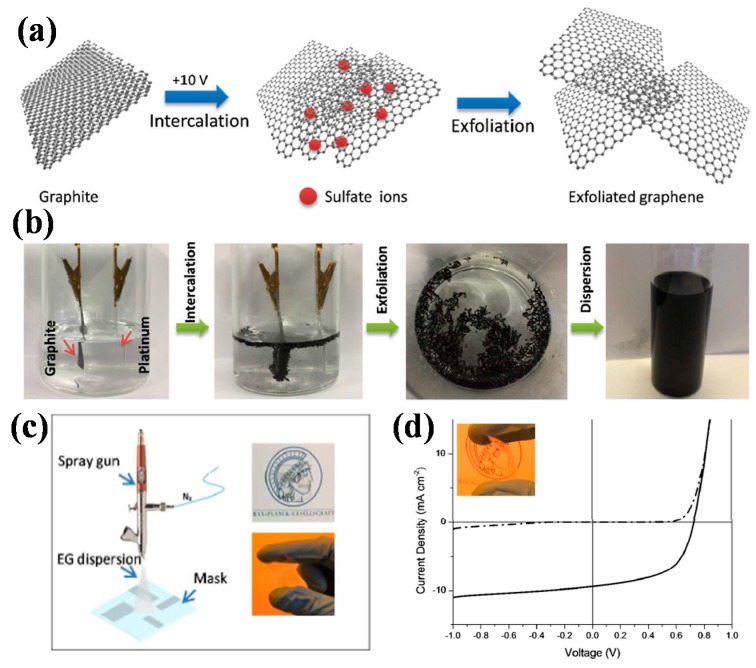
(**a**) Schematic illustration of the electrochemical exfoliation of graphite; (**b**) Optical images of the exfoliation process; (**c**) Schematic illustration of spray deposition of EG dispersion onto a substrate; and, (**d**) J–V characteristics of an EG-OSC on flexible substrate under light (solid line) and dark conditions (dashed line). Reprinted with permission from [62]. Copyright 2017 American Chemical Society.

Recently, Ricciardulli et al. fabricated graphene via an electrochemical exfoliation method, and applied it as transparent conductive electrode in polymer solar cells. The exfoliation process was shown in Figure 7a,b. The devices using PTB7:PCB71M as the active layer obtained PCEs of 4.23% and 3.77% on glass and PEN flexible substrates, respectively. The solution processed graphene electrodes got a relatively low sheet resistance of 0.52 kΩ/sq, with transparency of 70%. The graphene films were mechanically robust, without obvious change in *R*s at different bending angles. The resulting device was able to maintain 99% of the original efficiency after bending for 150 cycles. It was the first report using electrochemical exfoliated graphene as transparent electrode in polymer solar cells, this solution-based method contributed to the development of graphene based solar cells and other devices [62].

Other research aspects about the graphene electrodes include new transfer method of CVD-grown graphene [63], application of single-layer and fiber-shaped graphene [64,65], graphene meshes [66], graphene composites with carbon nanotubes, polymers, or inorganic nanoparticles [48,67,68], etc.

### 2.3. Metal Nanowires and Nanogrids

#### 2.3.1. Metal Nanowires

Metal nanowires, including Ag and Cu nanowires, are good alternative materials to ITO. Transparent conductive electrodes based on Ag nanowires (NWs) have attracted broad attentions, they were reported to exhibit good conductivity, light transmission, and mechanical flexibility, resulting in comparable solar cell performance to ITO-based devices [69,70].

Ag nanowires can be synthesized via several methods including photochemical reduction, electrochemical synthesis, template-assisted and polyol method, among which polyol synthesis is the most popular method [23]. The polyol synthesis typically involves the reduction of silver nitrate (AgNO_3_) by ethylene glycol with polyvinylpyrrolidone (PVP) serving as the stabilizer [71]. Through tuning the reaction parameters, such as the reaction temperature, molecular weight, and concentration of PVP, the addition of some salts, the size of Ag nanowires can be controlled [72,73]. As for the fabrication of Ag nanowires film as electrode, multiple methods can be adopted, including brush painting, spin coating, drop casting, spray coating, and so on. These solution based fabrication processes are cost-effective and simple, and are also applicable to flexible substrates.

Yang et al. fabricated Ag NWs film onto both ITO and flexible PET substrates through spray coating from Ag NWs solution, resulting in films with low sheet resistance (30.8 Ω/sq on PET substrate) and high transparency (80% from 400–2000 nm region). Flexible polymer solar cell using Ag NWs film as anode exhibited a PCE of 2.5% with device structure of Ag NWs/PEDOT:PSS/PBnDT-DTffBT:PCBM/Ca/Al. The open-circuit voltage (Voc) of the device was 0.3 V lower than the ITO based device, which could be ascribed to the low work function of Ag NWs/PEDOT:PSS film. Poor ohmic contact of the film with the upper active layer also led to a decreased FF, indicating that there was still room for improvement of the Ag NWs electrode. It is worth noting that the flexible devices based on Ag NWs electrode presented good mechanical stability, maintaining over 90% of PCE after bending up to 120° for 10 cycles [74].

**Figure 8:**
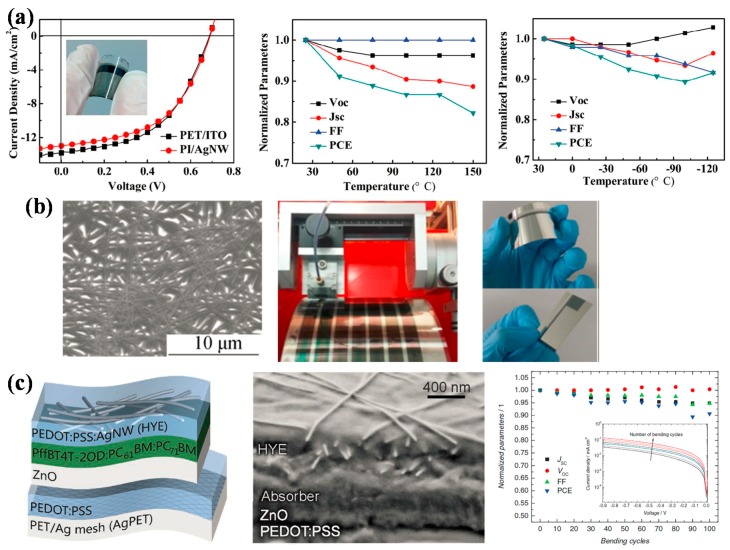
(**a**) J–V characteristics and normalized parameters of PI/Ag NWs based device during heating and cooling. Reproduced from [70] with permission of The Royal Society of Chemistry. (**b**) SEM of Ag NWs coated on PET substrate, fabrication of active layer in R2R process and flexible polymer solar cell with large-area (7 cm^2^). Reproduced with permission from [76]; published by Springer, 2016. (**c**) Device architecture, cross-sectional SEM image and evolution of the key performance parameters of the flexible polymer solar cell (>1 cm^2^) versus number of bending cycles. Reproduced with permission from [77]; published by John Wiley and Sons, 2016.

Surface roughness of Ag NWs film is an important issue concerning the performance of Ag NWs based solar cells, bare Ag NWs film tends to have a rough surface which hinders the improvement of device performance. Yu et al. reported the fabrication of stacked Ag NWs-polymethacrylate composite electrodes, the stacked Ag NWs contained a layer of short Ag NWs on the surface and a layer of long Ag NWs embedded inside. Flexible polymer solar cells that were based on the composite electrode with P3HT:PCBM as active layer achieved a PCE of 3.28%, similar to that of ITO glass based devices [69]. Through fabricating Ag NWs/polyimide (PI) composite structure, very smooth electrode surface was obtained, as demonstrated by Guo et al. The PI/Ag NWs composite electrode achieved excellent optical and electrical properties with transmittance of 83% and sheet resistance of 20 Ω/sq. What is more, the composite electrode presented outstanding resistance against thermal annealing, solvent exposure, repeated bending, tapping, or wiping, showing great mechanical, thermal, and chemical stability. It was also stable under low temperature down to −150 °C. Polymer solar cells using this flexible composite electrode achieved a PCE of 4.58%, close to that of the PET/ITO based devices (Figure 8a) [70].

The work function of electrode is also an important factor determining solar cell performance. Wang et al. demonstrated that the work function of Ag NWs film could be tuned through UV-ozone treatment. It was shown that after being treated by UV-ozone for 10 s, the work function of the Ag NWs film was increased from 4.3 to 4.9 eV, forming a more favorable band alignment, this increase could be attributed to the formation of silver oxide. After treatment, the Ag NWs electrode also exhibited higher transparency and conductivity. As a result, solar cell efficiency based on Ag NWs anode was improved from 0.76% to 1.73%. The efficiency was further improved by inserting PEDOT:PSS or MoO_3_ buffer layer between Ag NWs electrode and the active layer. PCEs of devices with PEDOT:PSS or MoO_3_ layer were 2.77% and 2.73%, respectively [75].

For practical applications, large area roll-to-roll process was usually preferred. Zhao et al. fabricated large-area (7 cm^2^) flexible polymer solar cells using Ag NWs as transparent conductive electrode. Ag NWs with different diameters were deposited on PET substrates through slot-die printing. The influence of Ag NWs diameter and slot-die coating times on the transmittance and resistance of Ag NWs film were investigated. The resistance could be controlled in the range of 73.9 to 6.4 Ω/sq. Photovoltaic devices with architecture of PET-Ag NWs/ZnO/PPDT2FBT:PC_71_BM/MoO*_x_*/Ag achieved a PCE of 1.87% without thermal annealing, and increased to 3.04% on the condition that the active layer was coated on substrate at 40 °C. This efficiency was much lower than that of the small area device, ascribing to the increase of resistance with device area scaling up. The roll-to-roll fabrication process and the resulting polymer solar cells were shown in Figure 8b [76]. An all solution deposition process was brought up by Czolk et al., using highly conductive PEDOT:PSS, combined with Ag NWs as top electrode and Ag mesh/PEDOT:PSS as bottom electrode. The device had a structure of PET/Ag mesh/PEDOT:PSS/ZnO/PffBT4T-2OD:PC_61_BM:PC_71_BM/PEDOT:PSS-Ag NWs (Figure 8c). Except for the printed Ag mesh, other layers were fabricated through doctor blading process, and a high PCE of 5.9% was obtained for the devices with active layer area over 1 cm^2^ [77]. The fabrication process demonstrated in these articles could provide some guidance for the industrial application of Ag NWs electrode in large-area polymer solar cells.

In the recent two years, the efficiency of polymer solar cells based on Ag NWs electrode has improved obviously, especially for the rigid devices that re fabricated on glass substrates. Wu et al. demonstrated a multi-length scaled Ag NWs grids based polymer solar cell with a PCE as high as 9.02%. The Ag NWs grids were fabricated by patterning of Ag NWs film on glass substrates. When compared with the original Ag NWs film, the patterned Ag NWs grids electrode exhibited improved optical transmittance. The resulting devices using Ag NWs grids as transparent conductive electrode (TCE) also outperformed the Ag NWs devices, close to the ITO counterparts. Device structure was demonstrated to be Glass/TCE/ZnO/PTB7-Th:PC_71_BM/MoO*_x_*/Ag [78]. A tandem device structure using Ag NWs as bottom electrode and interconnecting layer were reported by Raïssi et al., getting a PCE of 9.23%. Device structure was Glass/Ag NWs/ZnO/P3HT:ICBA/PEDOT:PSS/Ag NWs/ZnO/PTB7:PC_71_BM/MoO_3_/Ag, all of the layers were prepared through spin-coating, except for the top Ag electrode [79]. Solution processed method is favorable in large-scale industry production. Maisch et al. demonstrated a fully inkjet-printed polymer solar cell with Ag NWs as both bottom and top electrodes. This printing process using industrial printing heads could obtain Ag NWs films with good optical transmittance and conductivity, comparable to that fabricated via conventional slot-die or spray-coating method. The corresponding devices achieved a PCE of 4.3% with structure of Glass/Ag NWs/ZnO/PV2000:PC_70_BM/PEDOT:PSS/Ag NWs, and a device area of 1 cm^2^ [80]. The above results got on rigid glass substrates can provide some guidance for the fabrication of flexible devices.

**Figure 9:**
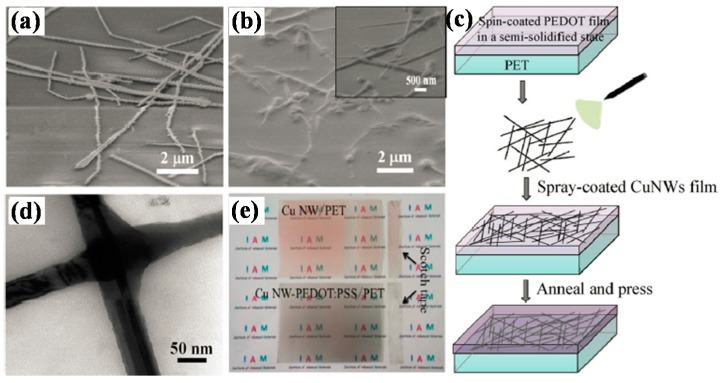
SEM images of (**a**) Cu NWs/PET and (**b**) Cu NWs–PEDOT:PSS/PET films; (**c**) Preparation procedure for Cu NWs–PEDOT:PSS/PET films; (**d**) TEM image of PEDOT:PSS-coated Cu NWs; (**e**) Photographs of adhesion test for Cu NWs/PET and Cu NWs–PEDOT:PSS/PET films. Reproduced with permission from [87]; published by Springer, 2015.

Cu nanowire is another kind of metal nanowire that has been adopted as electrode in flexible polymer solar cells, though it is not so broadly studied as Ag nanowire. Cu also has a high conductivity, close to Ag, and its advantage over Ag is the lower price and its abundance [81]. Fabrication and properties of Cu NWs based transparent electrodes have been studied, especially thin and long nanowires with high aspect ratio [81,82,83,84]. For Cu NWs electrode, the inter-wire contact quality is an important factor affecting the conductivity. Wang et al. developed a room temperature hydrogen plasma treatment method that could clean the surface and selectively weld junctions of Cu NWs networks, resulting in NWs films with low sheet resistance (19 Ω/sq) and high transparency (90%). Polymer solar cells based on the plasma treated Cu NWs electrode got a PCE of 2.67%, superior to the device using thermal annealed Cu NWs electrode [85]. Forming composite electrode could get improved optical-electrical properties when compared with bare nanowire films. As demonstrated by Kholmanov et al., the rGO/Cu NWs hybrid film had better conductivity, substrate adhesion, and stability than pure Cu NWs film [86]. Chen et al. fabricated Cu NWs-PEDOT:PSS composite film through a solution process, and applied it as transparent electrode in polymer solar cells. Cu NWs were spray-coated on PET/PEDOT:PSS film, followed by pressing at 20 Mpa to form a smooth surface, the nanowires were nearly completely buried in PEDOT:PSS (Figure 9). After optimization, the composite transparent electrode with a transmittance of 76% (at 550 nm) and a low sheet resistance of 15 Ω/sq was obtained. The resulting polymer solar cells with P3HT:PCBM active layer achieved a PCE of 1.4% [87].

#### 2.3.2. Metal Nanogrids

Metal nanogrids or nanomeshes have attracted extensive research interests in recent years as promising alternatives to conventional ITO electrode. Through tuning the grid or mesh line width, depth, and spacing, their optical and electrical properties can be controlled. Metal nanogrids using different metallic materials have been reported, including Ag, Au, Pt, Al, and so on. Among all of the materials, Ag is the one most studied, which is also the focus of this part.

**Figure 10:**
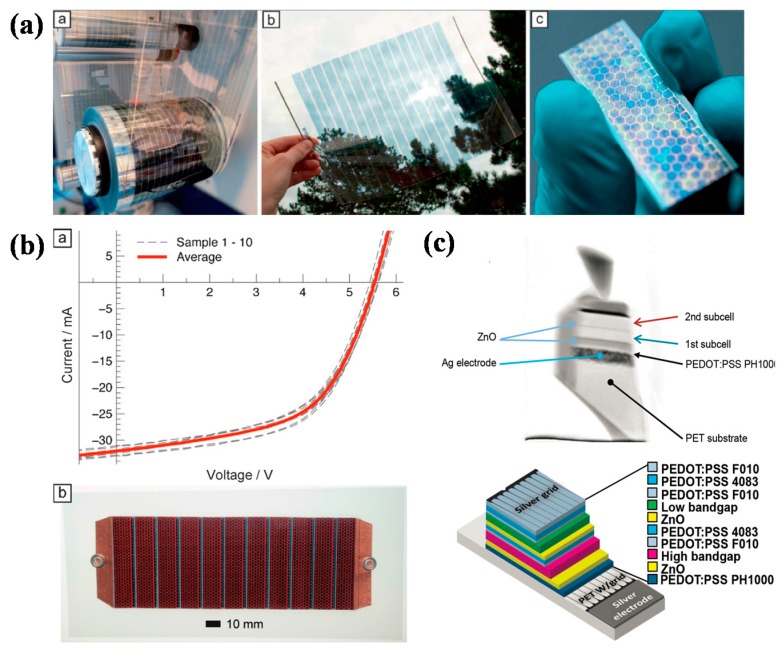
(**a**) Hundreds of meters of flextrode substrate (PET) ready for further R2R processing, flextrode substrate with 16 individual electrode stripes and mall flextrode cut-out suitable for OPV manufacturing using spin coating. (**b**) I–V curves (dashed lines) of ten randomly selected OPV modules containing eleven serial connected cells. The red line illustrates the average I–V curve. Fully encapsulated OPV module fabricated on the flextrode substrate. Reproduced with permission from [88]; published by John Wiley and Sons, 2013. (**c**) Ptychographic phase contrast projection of the polymer tandem solar cell stack, and schematic layout of the stack, showing the required layers for a full working cell. Reproduced with permission from [91]; published by John Wiley and Sons, 2015.

Frederik C. Krebs and coworkers have done a series of work using Ag-grids as electrode in polymer solar cells. The Ag-grid electrode was prepared by flexographic printing from Ag nanoparticle ink or Ag paste. They demonstrated a roll-to-roll printing fabrication process of flexible polymer solar cells with structure of Ag-grid/PEDOT:PSS/ZnO/P3HT:PCBM/PEDOT:PSS/Ag-grid (Figure 10a) [88]. Hexagonal Ag-grid was printed from Ag nanoparticle ink and Ag-grid/PEDOT:PSS/ZnO layers served as the front electrode. This electrode had good optical and electrical properties, with a low sheet resistance, as 10 Ω/sq and high transmittance of 60% across the visible region. PCEs of over 1.8% and 1.6% were achieved for single cell and modules (Figure 10b), respectively. They have also reported a similar device structure that got a PCE of 1.31% using a compact coating/printing machine [89]. Based on the same machine, polymer solar cell modules formed by serial connecting of four devices with a total area of 8 cm^2^ were fabricated. The device structure was the same as previously reported, except that four different polymers were adopted, including P3HT, PDTSTTz-4, PBDTTTz-4, and PV-D4610. The PCEs exceeded 3% for PDTSTTz-4 and PV-D4610 based devices. Solar cell stability over time and under illumination was investigated [90]. The scalable printing and coating process was also adopted to fabricate tandem solar cells on flexible substrates, achieving a PCE of 2.67%. An in-situ X-ray structural characterization technique was used during the fabrication process, allowing for a three-dimensional (3D) reconstruction of each layer in the solar cell (Figure 10c) [91].

**Figure 11:**
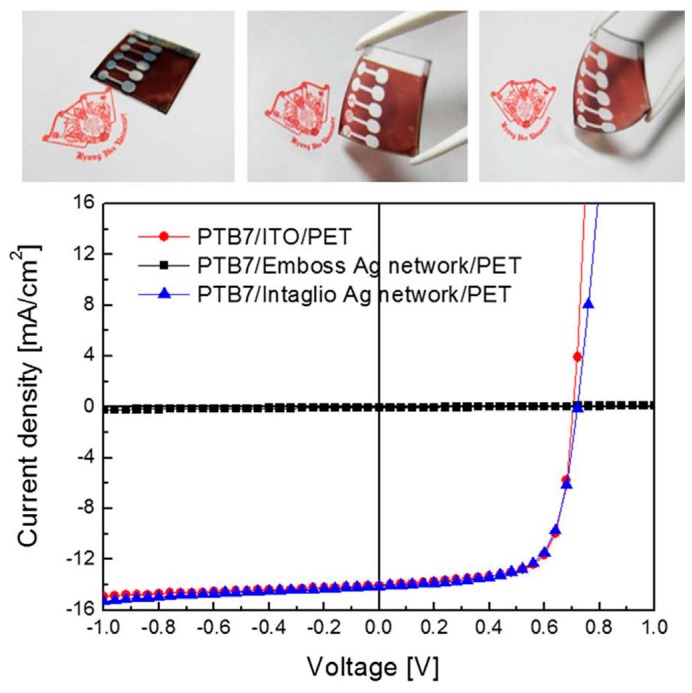
J–V curves of the flexible polymer solar cells based on ITO, emboss and intaglio-type Ag network electrodes. The upper pictures show the flexibility of the device using intaglio-type Ag network electrodes. Reprinted from [93], Copyright 2016, with permission from Elsevier.

The Ag-grids can be fabricated by direct printing on planar substrates as discussed above, they can also form an intaglio type by embedding the grids into substrates. The embedded structure is more likely to get high efficiency, as supported by several reports [92,93,94]. Seo et al. fabricated two kinds of mesh-like Ag networks with embossed and intaglio structure, respectively. Ag networks were formed by self-assembly of Ag nanoparticles that were coated on PET substrate via Meyer rod coating system. For the intaglio-type networks, a subsequent thermal transfer process was needed. Performance studies showed that both the embossed and intaglio networks exhibited similar transparency, conductivity, and flexibility, but the photovoltaic performance using these two networks as electrodes differed significantly, as shown in the J–V curves (Figure 11). The intaglio device had a high PCE of 6.955%, while the embossed one had a very low PCE as 0.436%. The poor performance was caused by nonuniformity of organic layers on embossed Ag network [93]. Ignasi et al. have also done some work to compare the photovoltaic performance of polymer solar cells using embedded and unembedded Ag-grids as anodes, and got consistent results with Seo, with the device based on embedded Ag-grids having a higher PCE than the unembedded one [94]. A smooth electrode surface is critical for high device performance. By coating UV-resin and high conductive PEDOT:PSS onto rough Ag-mesh, electrode film with low roughness was obtained, which was a guarantee for good performance. Ag-mesh was fabricated through a gravure-offset printing process, and UV-resin and high-conductivity PEDOT:PSS were coated subsequently. As a result, a solar cell efficiency of 6.9% with a high FF of 67.11% was demonstrated [95].

**Figure 12:**
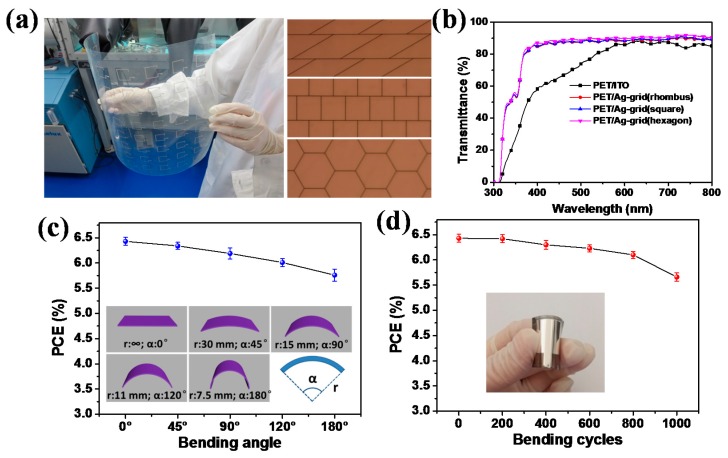
(**a**) Photograph of large-area PET/Ag-grid substrate (**left**), optical microscope images of three Ag-grid: rhombus, square and hexagon (**right**); (**b**) Transmittance spectra of PET/ITO and PET/Ag-grid (rhombus, square and hexagon) substrates; (**c**) PCEs measured while bending the flexible devices at different angles, the inset shows the schematic images of different bending angles (bending radii); and, (**d**) PCEs measured after bending the flexible device for different cycles with bending angle of 120°, inset shows the photograph of one flexible solar cell. Reprinted from [96], Copyright 2017, with permission from Elsevier.

We also used embedded Ag-grids as transparent electrode in our recent work, and achieved a high PCE of 6.51% for large-area (2.25 cm^2^) flexible polymer solar cells. PET/Ag-grid substrates were prepared using nano-imprinting technique, which was applicable for large scale fabrication. The key fabrication procedure for the obtaining of this high efficiency is the adoption of a modified buffer layer. By adding additives into PEDOT:PSS, compact contact was formed between PET/Ag-grid substrate and PEDOT:PSS buffer layer. From the viewpoint of the real application of polymer solar cells, mechanical flexibility is a major concern. We investigated the device stability against bending, and the result showed that our devices could retain 90.5% and 88.3% of the original PCEs under a bending angle of 180° and bending cycles of 1000, showing excellent flexibility (Figure 12). We also investigated the effect of Ag-grid patterns on device performance, the photovoltaic parameters showed little difference for rhombic, square, and hexagon Ag-grid based devices, ascribing to the similar transmittance of the substrates, the result indicated that it was the cover ratio, rather than the patterns, which had some effect on solar cell performance [96]. Modifying PEDOT:PSS layer can also tune the work function, as reported by Wang et al. Through doping PEDOT:PSS with polyethylenimine and ammonia aqueous, the work function of PEDOT:PSS layer could be decreased, thus minimizing the work function mismatch between the PEDOT:PSS and ZnO layers. As a result, S-shaped J–V curve was diminished and a PCE of 6.58% was obtained [97].

Sheet resistance of the flexible electrode is a critical parameter determining the solar cell efficiency, especially for large area devices. Mao et al. fabricated flexible polymer solar cells with an area of 1.21 cm^2^, using Ag grid/PEDOT:PSS(PH1000) hybrid electrode and got a PCE of 5.85% [98]. They investigated the performance of solar cells with different areas both for the Glass/ITO and PET/Ag-grid/PH1000 based devices. For small area (0.09 and 0.36 cm^2^) devices, the PCEs of ITO devices were higher than the Ag-grid/PH1000 ones, but when the device area was enlarged to 1 cm^2^, the latter showed better performance. This could be attributed to the low sheet resistance of Ag-grid/PH1000 electrode in a large area, the resistance of the ITO devices increased sharply while the Ag-grid/PH1000 stayed almost stable with the device area scaling up. Moreover, the PET/Ag-grid/PH1000 electrode has a superior mechanical flexibility over the PET/ITO electrode, as demonstrated by Li et al. [99]. The sheet resistance of Ag-grid/PH1000 kept below 2 Ω/sq, while that of the PET/ITO electrode increased from an initial 35 Ω/sq to over 1000 Ω/sq after bending at a radius of 3 mm. After bending for multiple times, the Ag-grid/PH1000 electrode was much more stable than the PET/ITO one.

In general, the efficiency of polymer solar cells based on Ag grid or mesh electrode has shown obvious improvement in the recent years [96,97,98,99,100,101,102]. Besides, other metal nanomeshes have also been reported as transparent electrodes, including Au, Al, Pt, Cu, etc. Au has better chemical stability than Ag, and it does not have the problem of oxidization. Guo et al. fabricated Au nanomesh using grain boundary lithography method, the Au nanomesh electrode exhibited excellent conductivity, transparency, and stretchability, which was suitable for application in flexible photoelectric devices [103]. Zhu et al. adopted Au nanomesh as anode in polymer solar cells with P3HT:PCBM active layer, and obtained a PCE of 3.12% [104]. When compared with noble metals like Au and Ag, Cu is cheaper and abundant. Flexible polymer solar cells using Cu nanowire mesh electrode was able to obtain a comparable PCE with ITO based devices, while showing much better flexibility [105]. Pt and Al nanomesh electrodes were also reported to have good optical and electrical properties, with both of them being fabricated while using anodic aluminum oxide (AAO) template [106,107]. A AgNi nanomesh electrode was demonstrated by Kim et al., showing higher durability than pure Ag nanomesh, which was due to a protective effect by nickel [108]. In summary, both metal nanowires and nanogrids electrode have been successfully applied as transparent electrode in polymer solar cells and good photovoltaic performance has been achieved even with a large device area. Some representative results are summarized in Table 1.

### 2.4. Conductive Polymer

Conductive polymers are another kind of promising alternative material for transparent electrodes, with PEDOT:PSS being the most successful and widely used polymer [109]. PEDOT:PSS is composed of conductive PEDOT and insulating PSS. PEDOT is not soluble by itself, but with the addition of PSS, it can be dispersed in water. By tuning the PEDOT to PSS ratio, the conductivity of PEDOT:PSS can be varied in a large range, making it appropriate for different applications [110]. Increasing PSS ratio will lead to decreased conductivity when considering the insulating nature of PSS. Heraeus has developed multiple grades of PEDOT:PSS with different conductivity. Clevios PVP AI 4083, with PEDOT to PSS ratio of 1:6, has conductivity in the range of 10^−3^ to 10^−4^ S/cm. It is commonly used as hole transporting material in organic solar cells. When used as electrode, a much higher conductivity is demanded. Highly conductive PEDOT:PSS, including PH500, PH510, and PH1000 has a PEDOT to PSS ratio of 1:2.5 and conductivity in the range of 0.2–1 S/cm [111]. It has attracted extensive attention in past several years, due to its excellent properties including high transparency, conductivity, stability and ease of fabrication, making it suitable as transparent electrode in flexible polymer solar cells [109].

Zhou et al. used PH500/PEDOT (EL) as anode in flexible polymer solar cells, and got an efficiency of 2.2%, which was 80% of that for ITO glass based devices [112]. They also fabricated multifolded devices consisting of four cells in series, the folded devices with an optimized opening angle of 30° could get 60% efficiency enhancement when compared with the planar devices [113]. In the recent years, more conductive PH1000 has substituted PH500. When compared with ITO, the conductivity of PH1000 is still low, thus improving the conductivity is critical for its application in solar cells, which is also the focus of research concerning PEDOT:PSS electrode. Several strategies have been applied to improve the conductivity, including adding some additives into PEDOT:PSS aqueous solution, post treatment of PEDOT:PSS films, etc.

**Figure 13:**
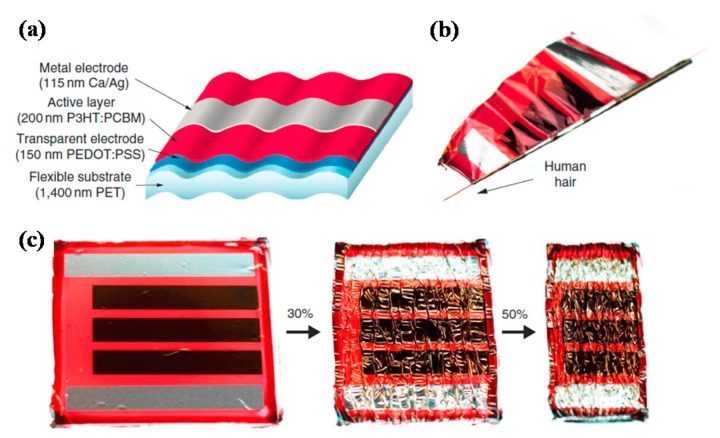
(**a**) Scheme of the ultra-light and flexible organic solar cell; (**b**) Extreme bending flexibility demonstrated by wrapping a solar cell around a 35-μm-radius human hair; (**c**) Stretchable solar cells made by attaching the ultrathin solar cell to a pre-stretched elastomer. Reproduced with permission from [114]; published by Nature Publishing Group, 2012.

Dimethyl sulfoxide (DMSO) is a well-known additive in PEDOT:PSS, which is also recommended by the supplier (Heraeus). Kaltenbrunner et al. used modified PEDOT:PSS as anode to fabricate ultra-thin polymer solar cells, the modified PEDOT:PSS was formed by adding 5 vol % DMSO and 0.5 vol % fluorosurfactant Zonyl FS-300 into PH1000 solution. The ultra-thin device had a total thickness of less than 2 μm, but achieved an equal PCE to their glass counterparts. To be noteworthy, the solar cells had a large specific weight of 10 W/g and could withstand severe mechanical deformation, as shown in Figure 13, providing directions for fabrication of ultra-thin, light, and flexible polymer solar cells [114]. Savagatrup et al. investigated the effect of the adding amount of DMSO, Zonyl and poly(ethyleneimine) (PEI) on the mechanical performance of PEDOT:PSS films, the result showed that adding 5% DMSO and 10% Zonyl was favorable for good mechanical property, while the highest conductivity was obtained by 0.1% Zonyl [115]. 

Post-treatment is another effective way to improve the conductivity of PEDOT:PSS films. Zhang et al. used a post-spin-rinsing method (PSRM) with DMSO to treat the pristine PEDOT:PSS films, getting a conductivity of 1335 S/cm, which was higher than that of films fabricated by adding 5% DMSO in the PEDOT:PSS aqueous solution before spin-coating. Polymer solar cells using the PSRM-treated PEDOT:PSS film as anode and PCDTBT:PC_71_BM as active layer obtained a PCE of 4.82% [116]. A conductivity as high as 4380 S/cm was achieved by sulfuric acid (H_2_SO_4_) post-treatment, which was comparable to that of ITO [117]. As a result, a similar photovoltaic performance was also achieved for polymer solar cells using H_2_SO_4_ treated PEDOT:PSS and ITO as anodes, respectively. This high conductivity could be ascribed to the structural rearrangement of PEDOT:PSS, forming highly crystalline nanofibrils. Though sulfuric acid is very effective in improving PEDOT:PSS film conductivity, there is a concern in real application that sulfuric acid is strong acid which may cause safety and environmental issues. Except H_2_SO_4_, various weak acids have also been adopted to treat the PEDOT:PSS film. As reported by Xia and Ouyang [118], weak acids were also able to improve the conductivity of PEDOT:PSS films, including sulfurous acid, acetic acid, propionic acid, etc. In the later work, Ouyang used a weak organic acid, methanesulfonic acid (CH_3_SO_3_H), to treat the PEDOT:PSS films and got a conductivity increase from 0.3 to 3300 S/cm, which was attributed to protonation effect of PSS, resulting in conformational change of PEDOT [119]. Fan et al. used modified PEDOT:PSS aqueous solution for film spin-coating, followed by methanesulfonic acid treatment, and got a high conductivity of 3560 S/cm. Flexible polymer solar cells using the PEDOT:PSS electrode got a PCE of 3.92%, which is close to that of ITO glass based devices [120]. They also developed a two-step mild acid treatment using methanesulfonic acid and phosphoric acid, combined with a film transfer method to fabricate PDMS/transferred PEDOT:PSS flexible electrode, and conductivity of 3560 S/cm was obtained. The flexible polymer solar cells with structure of PDMS/transferred PEDOT:PSS/PEDOT:PSS (4083)/PBDTT-S-TT:PC_71_BM/Ca/Al got a PCE of 5.38%. It was also demonstrated that mild acid treated devices presented a higher efficiency and better stability than H_2_SO_4_ treated devices [121]. Besides, there are other types of solutions or solvents that could get the conductivity of PEDOT:PSS films improved [122], and physical methods such as thermal and light treatment was also reported to be able to reach this goal [111]. A flexible all-plastic polymer solar cell using PH1000 as both the anode and cathode was demonstrated to reach a PCE of 2.88% [123].

### 2.5. Other Transparent Electrodes

Except for the well-known carbon nanotubes, graphene, metal nanowires, nanogrids, and conductive polymers, other types of transparent electrode in replacement of ITO have also appeared in the recent years. A representative one is ultrathin metal films, including Ag, Cu, and Au films [124]. Zuo et al. designed an ultrathin Ag film top electrode with thickness gradient, getting inhomogeneous conductance distribution [125]. This structure can realize energy loss reduction, which will facilitate the achievement of high polymer solar cell efficiency. By using this electrode, a high PCE of 7.15% was obtained for flexible polymer solar cell with a large area of 4 cm^2^. Stec et al. fabricated ultrathin (8 nm) Au films by thermal evaporation on glass, getting a film transmittance of over 80% in the range of 495–600 nm and a high conductivity of 11 Ω/sq [126]. Kang et al. reported a PEI/Ag/PEDOT:PSS hybrid electrode with a *R*s of 10 Ω/sq and high transmittance of over 95% in the visible range. Flexible polymer solar cells that were based on the hybrid electrode got a PCE of 10% [127]. Ultrathin metal films can be inserted between two transparent layers, forming oxide/metal/oxide (OMO) or dielectric-metal-dielectric (DMD) structure [124].

The conductivity of DMD electrode is mainly determined by metal layer, while the transmittance can be tuned by choosing appropriate dielectric materials [128]. Xue et al. employed nickel oxide (NiO) as the dielectric in DMD structure, forming a NiO/Ag/NiO (NAN) transparent electrode on flexible PET substrate. This flexible electrode exhibited good optical and electrical properties with transmittance of 77% in the visible range and sheet resistance of 7.6 Ω/sq. Polymer solar cells using the NAN electrode was fabricated with architecture of PET/NAN/PBDTTT-C:PC_70_BM/LiF/Al, a PCE of 5.55% was achieved, which is higher than that of PET/ITO based devices. Moreover, the stability of the PET/NAN based devices also surpassed that of the PET/ITO based ones [102]. Tungsten trioxide (WO_3_) is a commonly adopted dielectric in the DMD structure. WO_3_/Ag/WO_3_ electrode was reported to get a high optical transmittance of 81.4% together with a low sheet resistance of 19 Ω/sq [129]. A similar structure of WO_3_/Au/WO_3_ was used as bottom electrode in replacement of ITO [130,131]. There existed a microcavity resonant effect between Au and top Al electrode, which could enhance light absorption of active layers. The dielectric can be replaced by organic materials, for example, a poly(*N*-vinylcarbazole) (PVK)/Ag/PVK structure obtained a low sheet resistance of 10 Ω/sq with transmittance of 85% [132]. 

In the OMO structure, ZnO was normally adopted as the oxide layer. Flexible polymer solar cells using a modified ZnO/Ag/ZnO electrode as demonstrated by Zou et al., got a PCE higher than the ITO based devices [133]. Wang et al. replaced ZnO/Ag/ZnO with ZnO/AgO*_x_*/ZnO (O/Ag = 3.4 at %), getting an increased transmittance and a higher solar cell efficiency of 6.34% [134]. By mixing MgO with ZnO, Lee et al. reported an electrode structure of Mg*_x_*Zn_1−*x*_O/Ag/Mg*_x_*Zn_1−*x*_O, the optimal *x* is 0.28 and Ag thickness is 14 nm. The optimized electrode film has a Rs of 11 Ω/sq and an average transmittance of 89.2%, which is superior over the ZnO/Ag/ZnO electrode [135]. Ag can also be replaced by Cu, Zhao et al. fabricated a ZnO/Cu(N)/ZnO electrode using nitrogen doped Cu ultrathin film. This electrode has an average transmittance of 84% and a *R*s lower than 20 Ω/sq. It is worth noting that the resulting flexible polymer solar cells got a PCE of 7.1%, exceeding that (6.6%) of the ITO counterparts [136].

There are other structures that can acquire excellent optical and electrical properties, making them suitable for application as electrodes in polymer solar cells, for example, MoO_3_/LiF/MoO_3_/Ag/MoO_3_ [137], MoO_3_/Au/Ag/NPB [138], and MoO*_x_*/Ag/ZnS [139]. Polymer solar cells with high efficiency over 6% and device area as large as 25 cm^2^ were obtained [138,139].

## 3. Stability of the Flexible Polymer Solar Cells

As to the research of polymer solar cells, much attention has been paid to the improvement of device efficiency, as well as solution-based fabrication process. Selection of suitable flexible transparent electrodes and large scale roll-to-roll fabrication has also attracted great attention in the research area of flexible polymer solar cells. However, the stability issue, which is critical for real applications, has not been so much studied, with publications accounting for less than 5% of the total number of organic solar cells [140]. The stability of flexible polymer solar cells can be classified into two aspects, including mechanical stability and chemical stability [140]. Mechanical stability that was measured under different mechanical deformation conditions has received adequate attention in the recent years. In part 2 of this review, mechanical flexibility has been included in the discussion of each section of transparent electrodes. In this part, we will give a short discussion mainly about the chemical stability of polymer solar cells, in terms of material selection and encapsulation.

**Figure 14:**
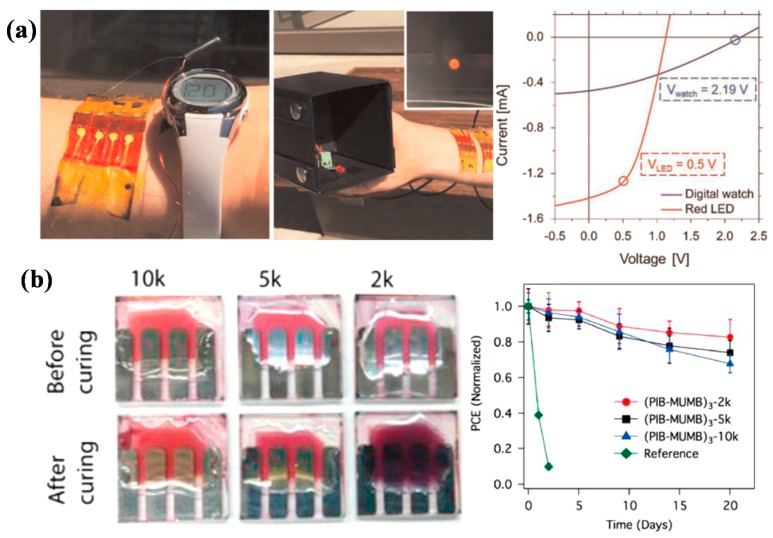
(**a**) Wearable organic solar cells on skin powering: a wearable digital watch in outdoor sunlight and an LED in outdoor sunlight (980 W·m^−2^), corresponding I–V characteristics of P3HpT:PCBM solar cells powering an LED(red) and digital watch(blue). Reprinted from [143], Copyright 2016, with permission from Elsevier. (**b**) Encapsulated device before and after photo-crosslinking and device stability in air. Reprinted from [140], Copyright 2016, with permission from Elsevier.

Son et al. investigated the effect of material selection of hole transporting layer (HTL) and fabrication method of the back electrode on the stability of polymer solar cells under damp heat conditions. The result showed that MoO_3_ hole transporting layer had better stability than PEDOT:PSS layer, and evaporated Ag back electrode had better stability than printed Ag electrode, which could be ascribed to the reaction between residual solvent in the Ag electrode and other layers. Thus, material and solvent selections should be taken into consideration in the research of polymer solar cells [141]. Beliatis et al. employed vanadium pentoxide (V_2_O_5_) to replace PEDOT:PSS as HTL, and studied the lifetime of the polymer solar cells using different encapsulation methods [142]. Except for the chemical stability, the selection of materials also has an effect on the mechanical stability, as reported by O’Connor and coworkers. They used poly(3-heptylthio-phene) (P3HpT) instead of the typical P3HT as electron donor material, resulting in largely improved mechanical stability. The corresponding solar cells were able to retain over 80% of the original PCE after 1000 cycles of compression. It is worth mentioning that the solar cells were adhered to human skin and were tested in ambient air (Figure 14a). This study provided some guidance for wearable organic electronics [143].

For the long-term application of the organic photovoltaics, encapsulation is very necessary to protect the devices from oxygen and moisture in the air [140,144,145,146,147]. By encapsulating flexible polymer solar cells with flexible barrier films, completely flexible devices could be obtained, and the devices were capable to retain over 95% of the initial efficiency after 1000 h damp heat ageing [144]. On one hand, the encapsulation process should be compatible with large area roll-to-roll fabrication process. On the other hand, the sealant that is used for encapsulation should have good stability. Highly flexible and stable materials are needed. Some polymer sealants, like polyvinyl alcohol and poly(methylmethacrylate), have unsatisfied stability over long time. Bag et al. employed polyisobutylene (PIB) as self-healing sealant, resulting in good device stability (Figure 14b) [140]. Edge sealing was also reported to have a great influence on the stability of polymer solar cell modules [119]. Both intrinsic properties of materials and encapsulation process affect the overall performance of photovoltaic devices or device modules.

## 4. Conclusions and Outlook

In this article, we have provided an overview on the research progress of ITO-free flexible polymer solar cells. The mostly studied alternative transparent electrodes in recent years are covered in this review, including carbon nanotubes, graphene, metal nanowires, and nanogrids, conductive polymers, etc. These electrodes can be either used alone or in combination with each other, and each has its own advantages as well as drawbacks. Sheet resistance and optical transparency are the two critical parameters that determine the quality of the electrode film. Taking practical application into consideration, the flexibility of the electrodes and the resulting devices and fabrication process are also very important aspects in the research of polymer solar cells. Some good results have been reported without the use of ITO, and the performance of ITO-free polymer solar cells has shown obvious improvement in the recent years. But currently ITO still dominates the market of transparent conductive films, and more research efforts are needed to be devoted to the area of alternative electrodes.

To be compatible with industry production, large area devices and roll-to-roll fabrication process will be a research trend in the future, and relevant research has already been conducted by some groups. Recently, flexible polymer solar cells with device area over 10 cm^2^ in tandem architecture were reported by Mao et al., and a high PCE of 6.5% was obtained [148]. Using the roll-to-roll method for the preparation of solar cells has also been reported to get good device performance [149]. The new concept, “water-borne polymer solar cells”, has been brought up and is likely to become a new hotspot in the future [150]. Recently, stretchable and waterproof organic photovoltaics with a high efficiency of 7.9% were reported by Jinno et al., pushing the development of wearable solar cells to a further step [151]. There are many possibilities for the development of ITO-free flexible polymer solar cells, we believe that the aforementioned transparent conductive electrodes will take place of ITO step by step, pushing flexible polymer solar cells closer to large scale practical application.

## Figures and Tables

**Table 1:** Photovoltaic performance of polymer solar cells based on metal nanowires and nanogrids electrodes.

Electrode	*R*s of electrode (Ω/sq)	Device structure	Device area (cm^2^)	Jsc (mA/cm^2^)	Voc (V)	FF (%)	PCE (%)	Reference
Ag NWs-polymer	10	Ag NWs-polymer/PEDOT:PSS/P3HT:PCBM/LiF/Al	0.14	9.71	0.52	65	3.28	[69]
PI/Ag NWs	20	PI/Ag NWs/PEDOT:PSS/PBDTTT-C:PC_70_BM/LiF/Al	0.12	13.01	0.69	51	4.58	[70]
Ag NWs	30.8	PET/Ag NWs/PEDOT:PSS/PBnDT-DTffBT:PCBM/Ca/Al	0.12	8.58	0.75	38.72	2.5	[74]
Ag NWs	36.5	PET/Ag NWs/PEDOT:PSS/P3HT:PCBM/LiF/Al	0.11	8.4	0.58	56.91	2.77	[75]
	36.5	PET/Ag NWs/MoO_3_/P3HT:PCBM/LiF/Al	0.11	8.4	0.58	56.07	2.73	[75]
Ag NWs	73.9–6.4	PET-Ag NWs/ZnO/PPDT2FBT:PC_71_BM/MoO_x_/Ag	7	10.45	0.71	40.6	3.04	[76]
Ag mesh and Ag NWs	16	PET/Ag mesh/PEDOT:PSS/ZnO/PffBT4T-2OD:PC_61_BM:PC_71_BM/PEDOT:PSS-Ag NWs	106–126	13.7	0.764	56	5.8	[77]
Ag NWs grids	28	Glass/Ag NWs grids/ZnO/PTB7-Th:PC_71_BM/MoO_x_/Ag	0.0863	17.8	0.78	65	9.02	[78]
Ag NWs	10	Glass/Ag NWs/ZnO/P3HT:ICBA/PEDOT:PSS/Ag NWs/ZnO/PTB7:PC_71_BM/MoO_3_/Ag	0.18	11.23	1.47	58	9.24	[79]
Ag NWs	18	Glass/Ag NWs/ZnO/PV2000:PC_70_BM/PEDOT:PSS/Ag NWs	1	10.7	0.76	52.8	4.3	[80]
Cu NWs	19	PEA/Cu NWs/TiO_2_/P3HT:PCBM/MoO_3_/Ag	NA	9.51	0.54	52	2.67	[85]
Cu NWs	15	PET/Cu NWs-PEDOT:PSS/P3HT:PCBM/Al	NA	6.05	0.58	40	1.4	[87]
Ag-grid	NA	PET/Ag-grid/HCPEDOT:PSS/ZnO/PDTSTTz-4:PCBM/PEDOT:PSS/Ag	8	2.1	2.72	55.5	3.2	[90]
Ag network	3.95	PET/Ag network/PEDOT:PSS/PTB7:PC_70_BM/Ca/Al	NA	14.11	0.718	68.60	6.95	[93]
Ag-mesh	13.26	PET/Ag mesh/H-PEDOT:PSS/PVP AI4083/PTB7:PC_71_BM/TiO_x_/Al	0.1143	14.21	0.73	67.11	6.94	[95]
Ag-grid	NA	PET/Ag-grid/ZnO/PFN/PTB7-Th:PC_71_BM/MoO_3_/Ag	2.25	14.30	0.773	58.14	6.43	[96]
Ag-grid	NA	Ag-grid/PEDOT:PSS/ZnO/PTB7-Th: PC_71_BM/MoO_3_/Al	NA	14.29	0.78	59	6.58	[97]
Ag-mesh	15	PES/Ag nanomesh/ZnO/PTB7:PC_71_BM/PEDOT:PSS/Ag	0.38	16.03	0.73	60.89	7.15	[100]

NA: not available.
